# 

**Published:** 1886-09

**Authors:** 


### ATTENTION.

With this number will be sent bills to those who are indebted for subscription. A few are in arrears for more than one year, though it is the aim of the publishers to cut off all who do not pay promptly. We respectfully but urgently ask an immediate response to these reminders. We have tried to serve our subscribers well, and now hope they will show their appreciation by helping to meet the necessary bills of the journal. Please send postal notes, postal orders, or drafts on New York. It costs too much to collect personal checks for such small amounts. Send all remittances to the Editor at Buffalo, N. Y.

